# Optimization of a pre-concentration method for the analysis of mercury isotopes in low-concentration foliar samples

**DOI:** 10.1007/s00216-023-05116-5

**Published:** 2024-01-09

**Authors:** Saeed Waqar Ali, Dominik Božič, Sreekanth Vijayakumaran Nair, Igor Živković, Jan Gačnik, Teodor-Daniel Andron, Marta Jagodic Hudobivnik, David Kocman, Milena Horvat

**Affiliations:** 1 Jožef Stefan Institute, 1000 Ljubljana, Slovenia; 2 Jožef Stefan International Postgraduate School, 1000 Ljubljana, Slovenia

**Keywords:** Foliage, Hg isotopes, Pre-concentration, Hg fractionation

## Abstract

Hg isotope analysis in samples from background regions is constrained by the presence of low Hg concentration and therefore requires a pre-concentration method. Existing Hg pre-concentration methods are constrained by long sample processing time and limited sample loading capacity. Using foliar samples as a test case, an optimized Hg pre-concentration method is presented that involves the microwave-assisted digestion of samples for Hg isotope analysis with the addition of a pre-digestion step. Microwave-digested foliar samples and CRMs were transferred to an impinger, reduced with SnCl_2_, and collected in a 2.25 mL concentrated inverse aqua regia (3:1 HNO_3_:HCl, v/v). This resulted in an optimal acid concentration in the solution ideal for analysis on MC-ICP-MS. The time for purging with Hg-free N2 was optimized to 30 min and the efficiency of the pre-concentration method was tested using a combination of approaches. Tests performed on pure reagents and matrix of foliar samples spiked with ^197^Hg radiotracer showed recoveries averaging 99 ± 1.7% and 100 ± 3.0%, respectively. Mercury at concentrations as low as 1.83 ng g^−1^ was pre-concentrated by digesting aliquots of foliage samples in individual digestion vessels. Recoveries following their pre-concentration averaged 99 ± 6.0%, whereas recoveries of 95 ± 4.7% and 95 ± 2.5% were achieved for NIST SRM 1575a (pine needle) and reagents spiked with NIST SRM 3133, respectively. Analysis using multicollector-ICP-MS showed low fractionation of δ^202^Hg during sample pre-concentration with no significant mass-independent fractionation. The proposed method is a relatively simple and robust way to prepare Hg samples for Hg isotopic analysis and is suitable even for complex biological matrices.

**Graphical abstract:**

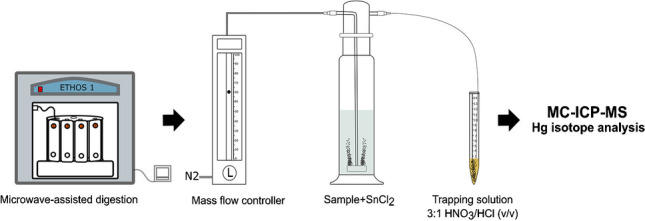

**Supplementary information:**

The online version contains supplementary material available at 10.1007/s00216-023-05116-5.

## Introduction

Recent advancements in inductively coupled plasma mass spectrometry (ICP-MS) enable the utilization of Hg stable isotopes to understand Hg biogeochemical cycling in the environment. Their application in various environmental compartments has allowed monitoring of changes in Hg sources, and their magnitudes. In this regard, an important utility of Hg isotopes is the acquisition of comparable data on the relative importance of Hg sources which is essential for global Hg monitoring programs and effective evaluation of the Minamata convention on mercury [1]. Stable Hg isotope signatures have provided new insights into transport and transformation processes [2], thus improving global Hg models to accurately estimate Hg pool sizes of major reservoirs and the magnitude of their intercompartmental exchanges [3]. In particular, the utilization of Hg isotope signatures has established forest ecosystems as a major sink for atmospheric Hg mainly via foliar Hg uptake and subsequent deposition to the forest floor via litterfall [4, 5].

Measuring Hg isotopes in samples is, however, constrained by the sensitivity of the analytical technique. Generally, the study of Hg isotopes in samples requires at least 0.5 ng g^−1^ of Hg in solution for analysis using a multicollector inductively coupled plasma mass spectrometer (MC-ICP-MS) with a sufficient level of certainty [6]. This is often challenging to achieve due to the low Hg concentration in samples. Foliar samples from sites having low anthropogenic influence tend to be at the lower spectrum of Hg concentrations [7]. This particularly relates to the foliar samples from the early spring season during which Hg concentrations are found to be the lowest, although Hg concentrations steadily increase across the season [8]. Studying Hg dynamics at background sites is, however, essential to understanding the Hg biogeochemical cycle, by exploring the natural variability of Hg in environmental reservoirs. In addition, background sites enable validation of existing Hg models and improve their accuracy for developing effective strategies to reduce Hg emissions and mitigate their impact on the environment [9]. The current effort is therefore aimed at addressing this by enabling Hg isotope analysis in foliar samples having low Hg concentration.

Several offline Hg pre-concentration methods exist [10–13] and among them is the widely used method that involves sample pyrolysis with dual-stage combustion followed by trapping of released Hg into a variety of oxidizing solutions [13]. The thermal-based reduction of solid samples at high temperatures has been shown to work ideally for a variety of samples with varying Hg concentrations. Solid samples are loaded in a quartz tube, plugged with quartz wool on both ends, and placed in the combustion furnace that reaches a temperature of 900 °C. Mercury-free O_2_ gas carries the combusted samples to the decomposition furnace that is held at 1000 °C before being purged into a trapping solution of inverse aqua regia (2:1, v/v) at 25 mL min^−1^ O_2_ flow rate. However, it takes 3.5 h to pre-concentrate Hg in one sample for Hg isotope analysis. Although this setup was further optimized for particulate matter (PM) samples collected on a quartz fiber membrane (QFM), the duration for Hg pre-concentration remained the same [11]. Sample loading in the dual-stage combustion method is further constrained by sample mass that may vary from 0.1 to 10g depending on the Hg concentration in the sample. The formation of soot as a result of incomplete combustion is linked with the carbon content which is often higher in samples such as coal or biomass. For instance, the combustion of more than 4 g of coal sample resulted in the formation of black deposits thus affecting the sample oxidation [13].

A similar pre-concentration method for Hg in solid samples involves hot acid digestion of samples before purging the digested solution onto a series of sample and analytical gold traps [10]. The loaded Hg on the gold traps is further desorbed for 40 min and captured in a variety of trapping solutions. The longer sample digestion time therefore does not make it suitable especially when working with a larger number of samples. To address this issue, the utilization of a pyrolysis unit for simultaneous detection of Hg in solid samples and Hg pre-concentration for Hg isotope analysis has also been proposed [12]. Analysis of certified reference materials (CRMs) using this method was statistically comparable to those reported previously with recoveries averaging 90 ± 4.0% based on certified values. This method offers a very short sample preparation time (8 min) although the amount of sample loaded is limited by the sample boat size. Pre-concentration methods involving purge and trap require the presence of Hg in a solution which is often challenging for samples with low Hg concentration as higher sample mass needs to be processed. While the existing offline pre-concentration methods are suitable for a wide range of environmental samples [10, 11, 13], Hg pre-concentration in environmental samples having low Hg concentration remains a challenge.

A few online Hg pre-concentration methods based on purge and trap systems have also been proposed [14–17]. The setup generally involves an online pre-concentration method using cold vapor generation and dual gold amalgamation coupled to MC-ICP-MS for Hg isotopic analysis. However, due to the transient nature of the output signal, precision loss may occur compared to continuous signal techniques. Additionally, the low signal-blank ratio for lower Hg mass introduced could potentially affect the accuracy of the method [15]. The online coupling of automated purge and trap with MC-ICP-MS for rapid and sensitive mercury isotopic analysis has been reported using a modified sample-standard bracketing (SSSSSB) sequence for isotopic measurements at ultra-trace levels without pre-concentration [14]. In such cases, the shorter integration time may lead to minor drift in Hg isotopic measurements. The potential of using chlorine-impregnated activated carbon (CIC) traps as an alternative to gold traps has also been studied [17], although there is a lack of information on the long-term stability and performance of the CIC traps over multiple uses or in varied environmental conditions. Conversely, modification of the arrangement of cones for enhanced signal sensitivity and optimization of instrument gas flows for steady and high Hg signal sensitivity have also been explored to obtain precise measurements of low Hg solutions [16]. In one study, an isotope binary mixing model was applied to calculate Hg isotopic compositions in low Hg concentration samples by mixing with a high-concentration Hg standard solution and using MC-ICP-MS for measurement [18]. The standard addition method was used to validate the precision and accuracy of the isotope data obtained from the binary mixing model. However, the study’s reliance on the standard addition method may not fully represent the complexity of different matrices found in natural samples.

Microwave-assisted digestion, on the other hand, involves the use of microwave radiation to facilitate the digestion of samples under analysis and offers application for a wide range of samples [19–23]. In addition to the rapid and efficient sample digestion under high temperatures and pressure, this method of sample digestion offers improved analyte sample recovery through complete decomposition of the sample. Consequently, this minimizes the matrix effect and interferences which is otherwise observed during thermal decomposition. Besides, the ability to process multiple samples under similar conditions enhances the reproducibility of results. However, samples with low Hg concentration particularly those from remote and low anthropogenically influenced regions require higher sample mass which subsequently restricts the use of a microwave-assisted digestion system. We, therefore, selected foliar samples with varying Hg concentrations to test the suitability of the optimized microwave-assisted digestion for sample preparation.

One of the main challenges of increasing sample mass with this method is the risk of strong reactions in the digestion vessels, leading to pressure build-up from the formation of gaseous NOx. Therefore, this requires the addition of a pre-digestion step where foliar samples react with reagents (HNO_3_, HCl, and H_2_O_2_) while allowing the generated fumes to escape. This enables higher sample mass to be completely digested while maintaining a high sample throughput. The pre-digestion step in addition to the versatility of microwave-assisted digestion therefore enables Hg pre-concentration and subsequently Hg isotope analysis in a wide range of samples. The current study therefore aimed at optimizing Hg pre-concentration in foliar samples as a test case. Furthermore, the aim was to validate the efficiency of the proposed pre-concentration method using ^197^Hg radiotracer and CRMs and investigate the associated Hg isotope fractionation during sample pre-concentration.

## Methodology

### Digestion

**Fig. 1:**
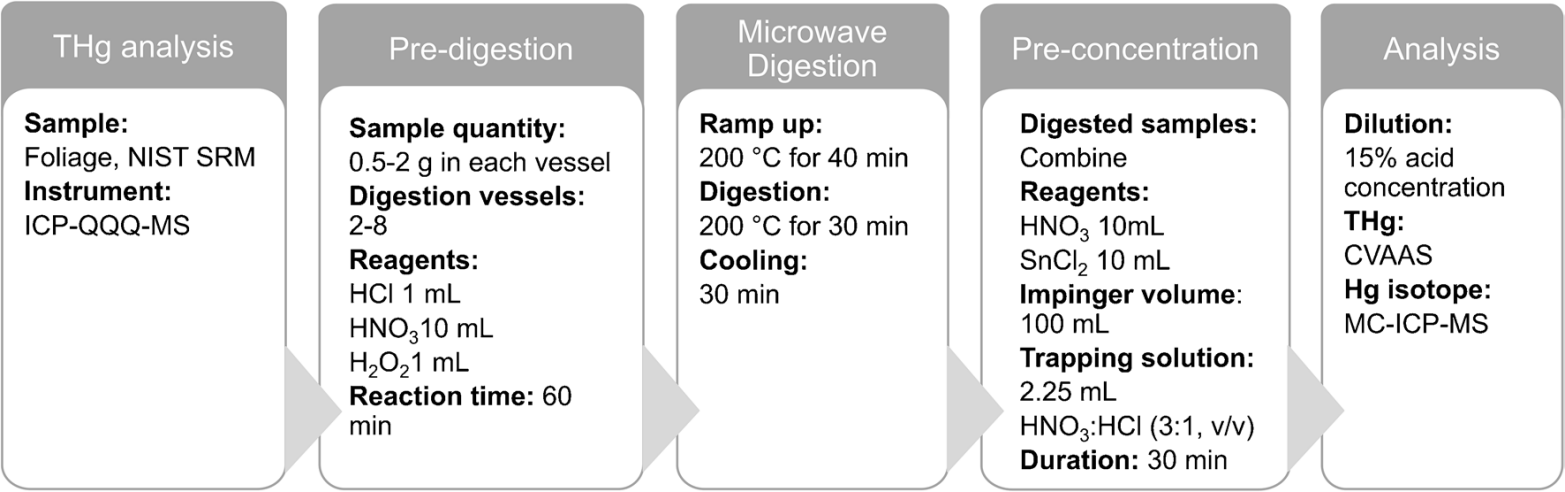
Schematic diagram of the proposed sample pre-concentration method

The proposed Hg pre-concentration method is schematically illustrated in Fig. 1. In the current study, up to 2 g of foliar samples was digested with the addition of 10 mL HNO_3_ (65% Suprapur), 1 mL HCl (37% Suprapur), and 1 mL H_2_O_2_ (37% Suprapur) using a microwave digestion system ETHOS 1 (Milestone Inc., Shelton, CT, USA). CRMs were digested in each batch along with foliar samples (*n* = 42) and procedural blanks. CRMs used in this study include NIST SRM 1575a (pine needles) (*n* = 14) and NIST SRM 1547 (peach leaves) (*n* = 2). Foliar samples were collected as part of the sample collection campaign under the ongoing GMOS-Train project (www.gmos-train.eu) to study Hg dynamics and fractionation in forests under various impacts in terms of Hg sources. The amount of foliar sample needed for digestion was determined by measuring Hg concentrations using a triple-quadrupole inductively coupled plasma mass spectrometer (ICP-QQQ-MS) (Agilent 8800, USA) following the method reported elsewhere [24]. Hg concentrations in foliar samples selected for this study ranged between 1.83 and 43.20 ng g^−1^ (*n* = 42) out of which 38% were below 10 ng g^−1^. The mass of the sample weighed in individual pre-cleaned Teflon vessels varied between 0.5 and 2 g. The concentration of foliar Hg was decisive for how much to weigh in each vessel. For instance, foliar samples that had Hg concentrations close to 2 ng g^−1^ were equally weighed in 8 vessels to have an equivalent of ~ 15 ng in the trapping solution. Samples having sufficient Hg concentration were weighed in 1–2 vessels. Aliquots of reagents were periodically added to the sample following the scheme shown in Fig. 1. Samples were first allowed to react with 10 mL HNO_3_ for 30 min before the addition of 1 mL HCl. Following a reaction time of 15 min, 1 mL H_2_O_2_ was added and allowed to react with the sample for a further 15 min. The generated fumes and pressure as a result of the reaction were frequently released while allowing the sample to react with the reagents.

Samples were subsequently digested in a closed vessel accompanying the microwave-assisted digestion under the following program: ramp-up time of 40 min, digestion at 200 °C for 30 min, and cooling for another 30 min. Following the digestion, the digested samples in the vessel were equilibrated at room temperature and samples were transferred into 50-mL vials and weighed. Before this, the initial weights of sample vials were recorded. Digestion vessels were also rinsed with high-purity MQ water (water with a resistivity of 18.2 MΩcm, purified in a Millipore Elix® Essential 5 UV Milli-Q system) and added to the sample vials making the total volume up to 20 mL. In this way, a maximum of 8 samples along with CRM and procedural blank were processed in a single batch of microwave-assisted digestion in less than 3 h.

### Pre-concentration

The setup for Hg pre-concentration included a 250-mL impinger connected to a 15-mL conical tube containing an oxidizing solution using a series of FEP (fluorinated ethylene-propylene) tubes of decremental sizes. Though purging Hg from a solution using impinger and pre-concentration in a trapping solution has previously been employed in a variety of schemes [10, 14, 25], the proposed purge and trap setup in this work is relatively simple and offers a robust method for pre-concentration, enabling efficient purging with a lower likelihood of Hg retention on the FEP tube walls. A Teflon adaptor with a press fit bore of 3.175 mm was connected to both openings of the impinger cap using an FEP tube having an inner diameter (ID) of 6 mm and an outer diameter (OD) of 8 mm. One end of the impinger was connected to a Hg-free N2 supply regulated by a mass flow controller (Cole Parmer) and the other end was further connected to a thinner FEP tube (0.8 mm ID × 1.6 mm OD) using an intermediate-sized FEP tubing (1.6 mm ID × 3.175 mm OD). The 15-mL conical tube contained 2.25 mL of a capturing solution prepared using inverse aqua regia (3:1, v/v) and was covered with Parafilm® M Sealing Film (Merck). The thin FEP tube (0.8 mm ID × 1.60 mm OD) was then inserted through the Parafilm and submerged into the capturing solution. The thin FEP tube (0.8 mm ID × 1.60 mm OD) was regularly purged with Hg-free N2 to remove possible droplets accumulated due to rinsing of the impinger. All connections were regularly checked for leakages using a portable gas leak detector (Restek Leak Detector, Restek Corporation, PA, USA).

The robustness of the proposed pre-concentration method along with the trapping efficiency of the inverse aqua regia (3:1, v/v) was evaluated. An acid concentration up to 30% v/v is tolerated for analysis using MC-ICP-MS. We, therefore, tested both diluted and concentrated inverse aqua regia (3:1, v/v) for optimal trapping efficiency at different flow rates and purging duration. This was done by spiking the solution in the impinger with an aliquot of NIST SRM 3133 and capturing it in the trapping solution. Hg in the trapping solution was subsequently measured with a cold vapor-atomic absorption spectrometer (CV-AAS) (Automatic Mercury Analyzer Model Hg-201, Sanso Seisakusho Co., LTD) using the method described previously [26, 27]. The instrument was calibrated using NIST SRM 3133 and peak heights measured for both sample and standard were used to calculate the concentration of Hg in the trapping solution.

### Validation of pre-concentration with ^197^ Hg radiotracer

We used a highly sensitive ^197^Hg radiotracer to validate the efficiency of our proposed pre-concentration method and to check the effect of the sample matrix during this process. The ^197^Hg radiotracer offers certain advantages for the validation of analytical methods. This is due to its high specific activity, relatively simple detection method, and free of external contamination as ^197^Hg is not present in nature. The process of ^197^Hg radiotracer production has been previously well described [28–30]. Following irradiation, the ^197^Hg stock solution was diluted to the desired concentration to create a working standard for the experiments allowing the evaluation of the efficiency and robustness of the proposed pre-concentration method.

An aliquot of ^197^Hg radiotracer working standard solution (Hg concentration 300 pg—30 ng in absolute mass) corresponding to 3–300 pg mL^−1^ was added to the impinger containing 100 mL of 10% HNO_3_ and 10% SnCl_2_ (v/v) and capped immediately. Considering the short half-life of the ^197^Hg tracer (2.671 days), the amount of the used working standard solution had to be increased since the experiments were performed on different days. Otherwise, the measured activity of the same ^197^Hg spike would be too low for reliable quantification using the gamma detector after several days.

The solution was then purged with N2 for 30 min following the Hg reduction with SnCl_2_ and ^197^Hg radiotracer was captured in the 2.25 mL inverse aqua regia (3:1, v/v). To evaluate the effect of the sample solution on pre-concentration efficiency, tests were carried out with digested foliage samples. Similar to the test with pure reagents, around 80 mL of digested samples was transferred to the impinger having 10% HNO_3_ and 10% SnCl_2_ (v/v) making a final acid concentration of 30% (v/v). The sample solution was then spiked with a known concentration of ^197^Hg radiotracer and captured similar to the test solutions with only pure reagents. As ^197^Hg is not present in nature, this approach offered a simple test for possible matrix interference during Hg reduction in the solution obtained from the digested sample.

The activity of ^197^Hg in solution was detected by a well-type HPGe detector (Model GCW6023/S), using the methods described previously [28]. Standards with concentrations corresponding to spikes (ranging from 300 pg to 30 ng) used in each experiment were prepared in triplicate (matching total volumes in the impinger and trapping solution) for the quantification of the ^197^Hg leftover in impinger and ^197^Hg recovery in the trapping solution, respectively. The corresponding activities of standards were measured in the detector and compared with the measured activities of samples (both concentrations in impinger leftover and recovered in trapping solutions). Mass balances are reported as the sum of corresponding leftovers in the bubbler and recoveries in the capture solution. The activity of ^197^Hg was determined by a peak area comparison of the characteristic doublet peaks of γ-ray and X-ray emissions (67.0 + 68.8 and 77.3 + 78.1 keV) using Genie 2000 Gamma analysis software.

### Isotopic analysis

Hg isotopic ratios in samples were measured using Nu Plasma II (Nu Instruments Ltd., Ametek, UK) MC-ICP-MS following the previously reported method [24]. Briefly, the system uses an auto-sampler ASX-520 (Teledyne Cetac, NE, USA) for sample introduction. SnCl_2_ in a solution (3% m/v in 10% v/v HCl) was used to reduce Hg, and then the mix of solutions was carried to the gas–liquid separator (Phase Separator Assembly, 2600 System, Model 2600-STD Tekran) connected to the torch using Teflon tubes resulting in a recovery as high as 99.6%. The elemental Hg vapor was transported by Ar sweep gas flow to the instrument, allowing for measurements of up to 1 ng mL^−1^. Intensities at m/z of 202 were between 0.5 and 1.2 V, and outlier rejection of individual measurements within the cycle was done.

To ensure consistency between published data, Hg isotope data obtained in the present study were reported using the sample-standard-sample bracketing technique [31], and outlier rejection of individual measurements within the cycle was done for values above the relative three standard deviations (SD). The results were reported using δ and ∆ notations [2]. Δ values were calculated from determined δ^xxx^Hg values using the expression: Δ^xxx^Hg ≈ δ^xxx^Hg – δ^202^Hg × f (where xxx denotes the mass number of the isotope), and correction factors f of 0.2520 for ^199^Hg, 0.5024 for ^200^Hg, 0.7520 for ^201^Hg, and 1.4930 for ^204^Hg. The measurement uncertainty is reported as long-term reproducibility of the measurements was evaluated by the NIST SRM 8610 (UM-Almadén) (National Institute of Standards Technology, 2017), and all the uncertainties are expressed at k = 2.

### QA/QC

ICP-QQQ-MS was used for the quantification of Hg concentrations in foliage samples. The limit of detection (LOD) reported (0.001 ng g^−1^) for the measured solutions was calculated as 3 times the standard deviation of procedural blanks divided by the slope of the calibration curve. The limit of quantification (LOQ) was calculated as 3 times the LOD and was 0.003 ng g^−1^. Hg concentration in all the analyzed samples was above the LOQ. Similarly, the LOD (0.03 ng) for CVAAS used to quantify Hg concentrations in the trapping solution was calculated as three times the standard deviation of the procedural blank (*n* = 12). The LOQ (0.1 ng) for CVAAS was calculated as 3.33 times the limit of detection. The quality assurance of the analytical method was achieved by analyzing NIST SRM 1575a (pine needle), NIST SRM 1547 (peach leaves), and procedural blanks in each batch of sample digestion step. Hg concentrations in CRMs and procedural blanks, similar to foliage samples, were determined using CVAAS following the pre-concentration step. CVAAS offers several advantages, particularly the ease of operation and shorter analysis time, which allows the instant analysis of Hg concentration in the trapping solution following the pre-concentration step. After each batch of digestion, the vessels were thoroughly rinsed with MQ water and a cleaning cycle was run with the addition of 10 mL HNO_3_ and 10 mL MQ. Digestion vessels were cleaned with a clean-up program (15 min ramping up to 200 °C and 30 min of ventilation). Blanks were then checked regularly for each digestion vessel before the next batch of sample digestion. When blanks higher than 0.10 ng corresponding to 5% of Hg in the trapping solution were observed, the contamination of the digestion vessels was checked with an absorbing microwave test. Empty digestion vessels were placed in the microwave digestion system for 1 min at 1000 W and checked for temperature rise. When the temperature of the digestion vessels exceeded 50 °C, an additional cleaning step was performed that involved heating the digestion vessels in a drying oven (SP 55-Easy, Kambic laboratory equipment) at 150 °C for 3 h. This ensured the release of the built-up fumes on the walls of the digestion vessels. This is usually necessary when a change in the color of the digestion vessel is apparent. Impingers used for pre-concentration were rinsed with MQ between each pre-concentration run.

## Results and discussion

### Mercury pre-concentration efficiency test

**Fig. 2:**
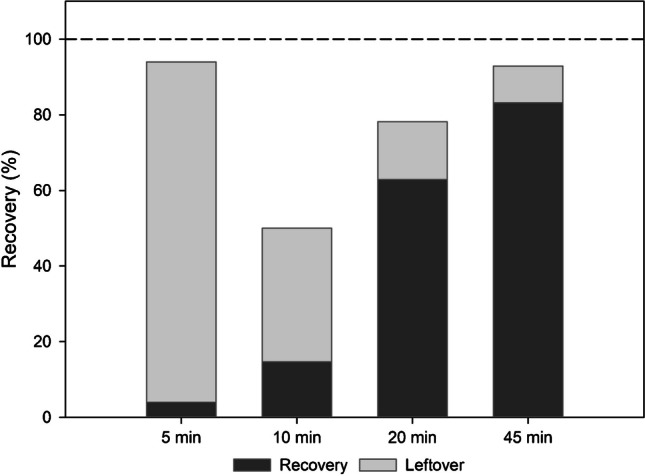
Hg trapping efficiency in 10 mL of 30% v/v inverse aqua regia (3:1 HNO_3_:HCl) at different purging durations

Trapping efficiencies of some of the common oxidants have been evaluated elsewhere and tests with 40% v/v inverse aqua regia using thermal desorption of Hg from gold traps have shown recoveries of 92 ± 4.0% and 98 ± 2.0% for 2 mL and 10 mL trapping solutions [10]. In the current work, tests on pure reagents spiked with 15 ng of freshly diluted NIST SRM 3133 (Hg standard solution), corresponding to 1 ng mL^−1^ in the trapping solution, were carried out. Hg trapped in 10 mL of 30% v/v inverse aqua regia solution showed increased recoveries with an increase in purging time thus indicating poor purging efficiency at a lower N2 flow rate (Fig. 2). Additionally, the analyte losses from the 30% inverse aqua regia trapping solution were also observed as the highest recovery achieved with this setup was 83 ± 2.0% for 45 min of purge time. Recovery subsequently did not increase with an increase in purging time thus displaying poor trapping efficiency of the 10 mL of 30% v/v inverse aqua regia, and therefore Hg isotope analysis was not performed. Results of poor trapping efficiency even with 40% v/v inverse aqua regia were also noted previously under similar N2 flow rate [12] and are more suited for long purging duration under lower N2 flow rate (5–50 mL min) [11, 13].

**Fig. 3:**
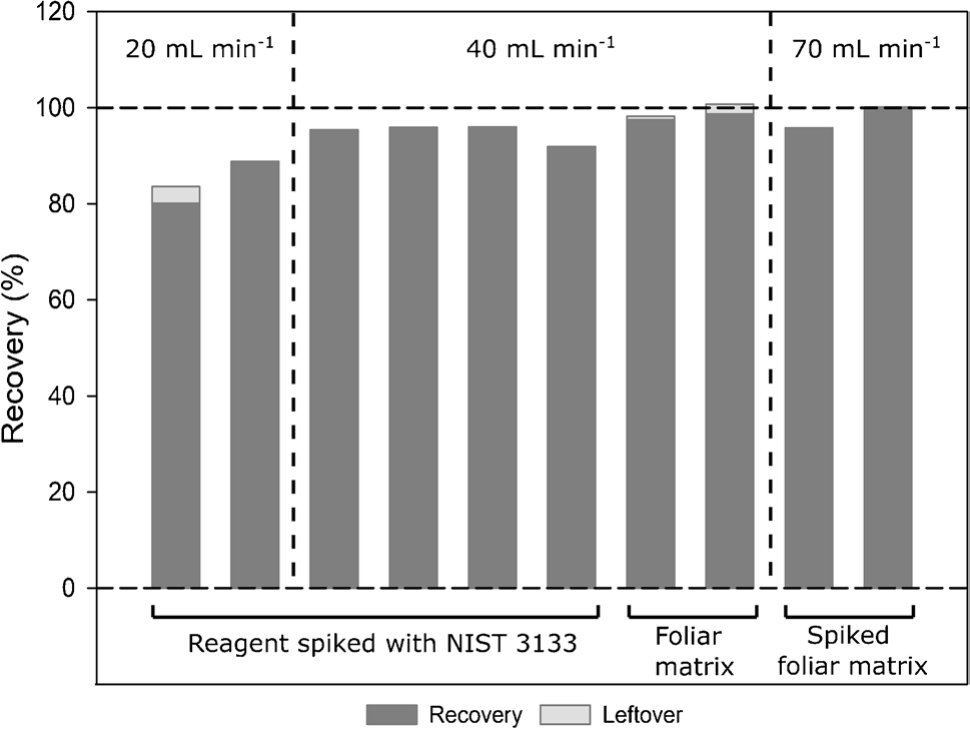
N2 flow rate influence and Hg recovery of NIST SRM 3133 (Hg solution) and digested sample solution using 4.5 mL of concentrated inverse aqua regia as trapping solution

Having observed poor trapping efficiency with the 30% v/v inverse aqua regia due to losses, a similar trapping efficiency test was then performed with 4.5 mL concentrated inverse aqua regia. Recovery improved significantly (Fig. 3) for 20- and 40-mL min^−1^ N2 flow rate for 45-min purge time with an average recovery of 84 ± 6.2% and 95 ± 1.9%, respectively. Further tests were carried out for the same purging time to check the effect of the leaf matrix on Hg pre-concentration efficiency. Recoveries of 98 ± 0.91% were observed for tests with microwave-digested sample solution constituting a nominal acid content of 32% (v/v) in the impinger (the acid content refers to the nominal volumes of nitric and hydrochloric acids used for the digestions of two leaf aliquots which were later mixed in the impinger for a single pre-concentration in a trapping solution). Recoveries for the Hg spiked to the sample solution, however, did not significantly improve with an increased N2 flow rate of 70 mL min^−1^. This indicated a strong trapping efficiency of the concentrated inverse aqua regia (3:1, v/v) even at a lower N2 flow rate. Since the acid content of the trapping solution after dilution with MQ water would amount to 30% of the solution for Hg isotopic analysis, further optimization tests were carried out to reduce the purge time and the acid content.

Subsequent tests involved pre-concentration of Hg into 2.25 mL of the concentrated inverse aqua regia (3:1, v/v) which would represent 15% acid content of the capturing solution when diluted to 15 mL. Having achieved more than 98 ± 0.91% recovery at 45 min of purging time during previous attempts, the aim was to lower the purging time which would significantly impact sample throughput. Therefore, the flow rate of Hg-free N2 was increased to 180 mL min^−1^ and Hg was captured in 2.25 mL of concentrated inverse aqua regia (3:1, v/v). With a purging time of 30 min, recoveries ranged from 91 to 100% with an average recovery of 95 ± 2.5% (*n* = 13) as shown in Table S4. The subsequent decrease in purging time resulted in lower recoveries and optimization of purging time was therefore not further pursued.

One study reported that recoveries as low as 50% did not cause significant mass-dependent fractionation [27], whereas recoveries between 81 and 102% have also been reported to show no systematic variation in Hg isotope signature for selected sample types [13]. However, recoveries close to 100% for Hg isotope analysis are warranted due to the possible loss of light or heavy Hg isotopes during Hg pre-concentration involving different steps [32]. We also tested trapping Hg from two impingers in a single trapping solution and recoveries with this setup averaged 93 ± 2.0% suggesting a significantly strong trapping efficiency of the concentrated inverse aqua regia even at a higher purging rate.

### Pre-concentration validation with ^197^Hg radiotracer

**Fig. 4:**
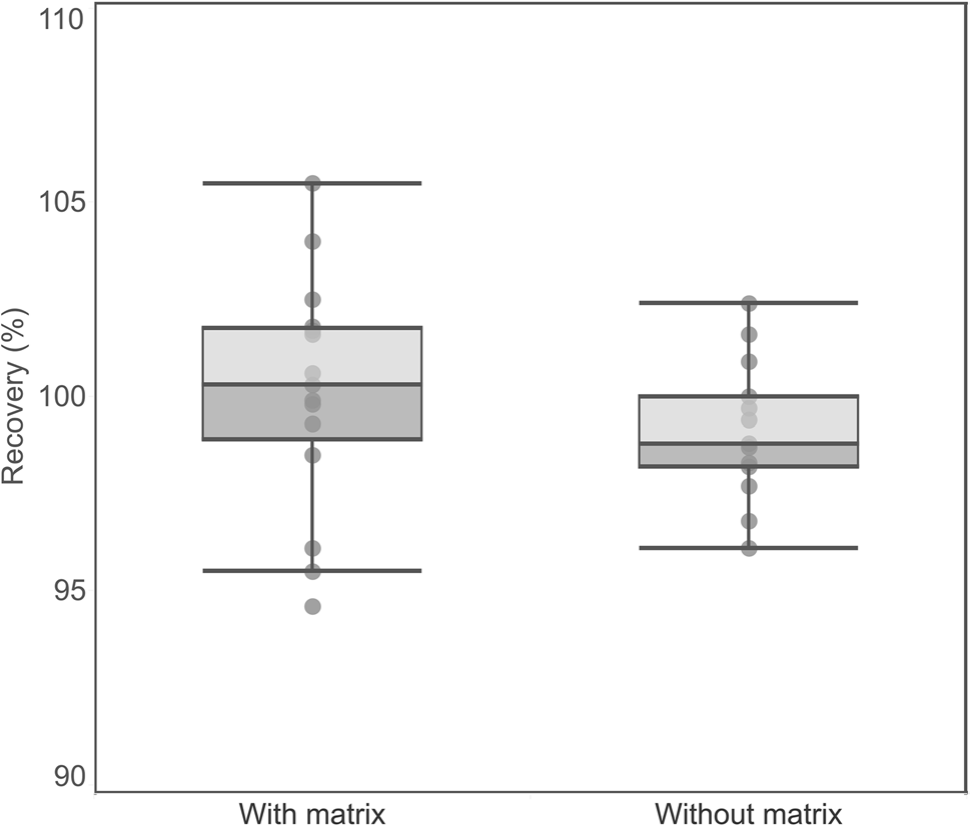
^197^Hg radiotracer recoveries for the effect of sample matrix on pre-concentration efficiency

Before Hg pre-concentration in solutions of digested foliar samples and CRMs, a validation test for the proposed Hg pre-concentration method was performed. This was required since working with low Hg concentration increases errors due to Hg contamination and Hg carryover between samples. Hg^2+^ is a highly reactive Hg species and may be retained on surfaces of sample containers, in this case, the impinger [33] which can be augmented by improper cleaning of the purging unit between samples. For this purpose, the highly sensitive ^197^Hg radiotracer is an ideal tool to validate our proposed pre-concentration method. Results from the ^197^Hg radiotracer experiment validated the pre-concentration efficiency of the pre-concentration method with an average 99 ± 1.7% recovery (Fig. 4). With recoveries ranging between 96 and 102% (Table S2), this indicated that Hg carryover between samples was minimal and did not interfere with the pre-concentration efficiency. The effect of the sample matrix on pre-concentration efficiency was also negligible though Hg pre-concentration in the presence of a sample matrix resulted in relatively higher recoveries compared to those performed without the addition of a sample matrix, they remain in the acceptable range of 94–105% recovery (Table S3).

### Pre-concentration of CRMs and foliar samples

Having validated the pre-concentration efficiency, Hg in solutions from foliar samples and CRMs was pre-concentrated and determined. With a pre-digestion step, up to 2 g of foliar samples having Hg concentration in the range of 7–8 ng g^−1^ could be digested in a single digestion vessel and thus did not require the merging of digested samples from multiple vessels into individual impingers but was needed for foliar samples with lower Hg concentration. For instance, foliar samples having Hg concentration in the range of 2 ng g^−1^ digested in 8 vessels were transferred equally into 2 bubblers and Hg pre-concentrated into a single trapping solution. This was done to maintain a sufficient acid concentration of solution in the impinger so that the Hg reduction efficiency by SnCl_2_ would not be affected. Procedural blanks in this case accounted for less than 5% of the lowest Hg concentration and 0.04% in the case of the highest Hg concentration in foliar samples used for the optimization of the pre-concentration method.

**Fig. 5:**
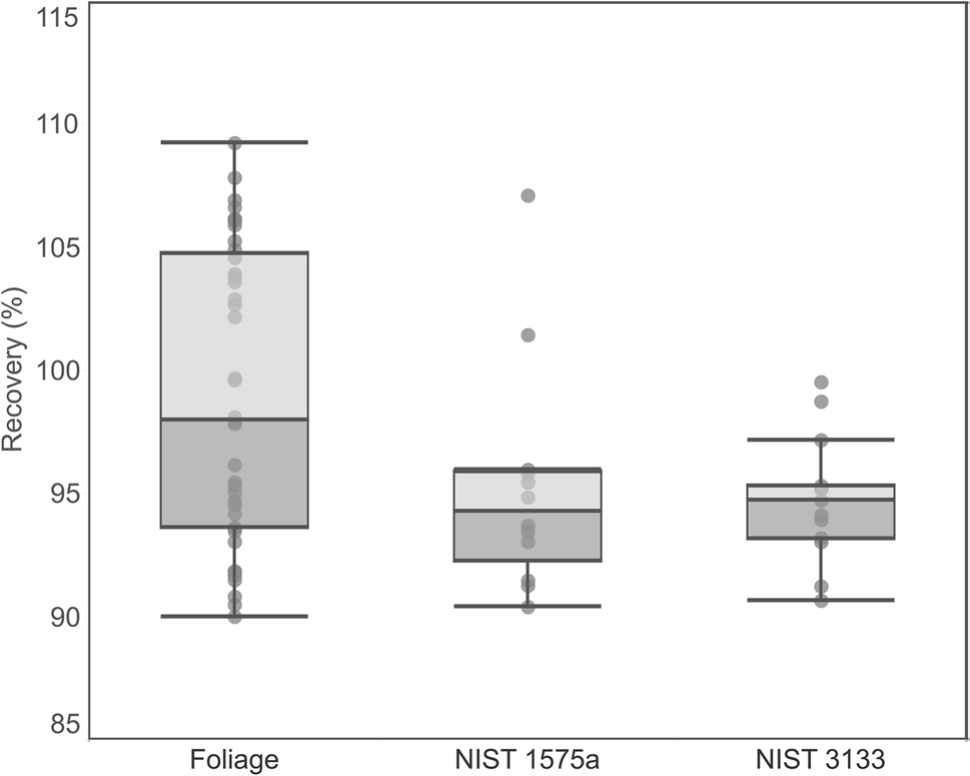
Recovery of Hg in foliar samples, pine needle (NIST SRM 1575a), and Hg standard solution (NIST SRM 3133)

Figure 5 summarizes the percent recoveries for foliar samples and CRMs. Recoveries for foliar samples were reported as the Hg concentration measured in the trapping solution relative to the original concentration determined in the samples using ICP-QQQ-MS. Recovery for foliar samples (*n* = 42) showed a relatively larger variation (99 ± 6.0%), although they were well within the acceptable range of 90–108%. The relatively wider variation in recoveries could also be due to the differences in homogeneity of studied foliar sample used in this study. We calculated the pre-concentration factors by dividing the absolute mass of pre-concentrated Hg (in ng) in the trapping solution with the Hg content in foliage samples (in ng/g). This factor (ranging from 0.77 to 10.70 g) is equal to the sample mass normalized to the procedural recovery. This factor could potentially be useful for comparison with other pre-concentration methods and for the estimation of the required sample weight when applying this method to other sample matrices in future studies. Recoveries for CRMs were on average 95 ± 4.7% for NIST SRM 1575a (pine needle) (*n* = 14) and 96 ± 5.6% for NIST SRM 1547 (peach leaves) (*n* = 2). These results therefore validated the robustness and efficiency of the proposed pre-concentration method.

### Isotopic fractionation during pre-concentration

**Fig. 6:**
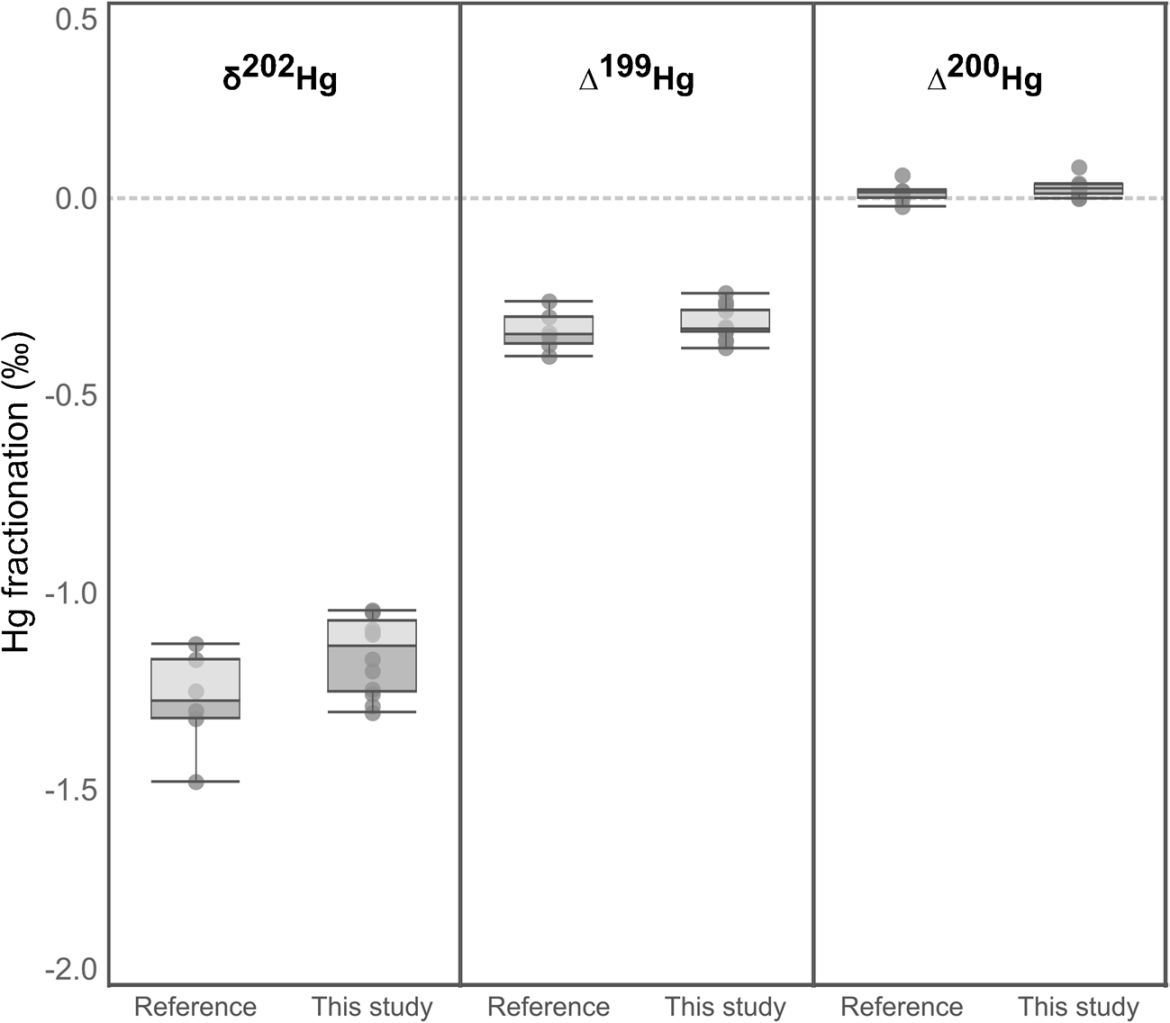
Hg fractionation in NIST 1575a (pine needle) compared with previously reported data. Data of reference values were acquired from reference [12, 18, 34–37]

Mercury isotopes in the analyzed CRM samples were measured using MC-ICP-MS and the results are summarized in Table S1. Long-term uncertainty was investigated through analysis of the secondary standard NIST SRM 8610 and all uncertainties associated with Hg isotopic data were reported as k = 2. Negligible δ^202^Hg (0.03 ± 0.15‰), ∆^199^Hg (− 0.02 ± 0.06‰), and ∆^200^Hg (0.00 ± 0.03‰) fractionation during Hg pre-concentration were observed for NIST SRM 3133 (Hg standard solution). The δ^202^Hg for NIST SRM 1575a (pine needle) was − 1.69 ± 0.33‰ whereas ∆^199^Hg and ∆^200^Hg were − 0.31 ± 0.08‰ and 0.02 ± 0.03‰, respectively. These results are comparable to previously reported values for this CRM [12, 18, 34–37] as shown in Fig. 6. Meanwhile, to our knowledge, Hg isotopic ratios have not been previously reported for NIST SRM 1547 (peach leaves). This study therefore reports − 1.72 ± 0.02‰, − 0.13 ± 0.01‰, and 0.01 ± 0.01‰ for δ^202^Hg, ∆^199^Hg, and ∆^200^Hg respectively for NIST SRM 1547 (peach leaves). Since the reference materials are not certified for Hg isotope signatures, our results demonstrate good agreement with values reported in the literature and validate the robustness of the proposed pre-concentration method.

## Conclusion

Analyzing Hg isotope signatures is challenging in foliar samples having low Hg concentration due to higher sample mass requirements. This work therefore proposes an optimized Hg pre-concentration method using the lowest foliar sample mass needed. Currently, no existing method is suitable for such low Hg concentration foliar samples. The method enables Hg isotope measurements in low Hg concentration foliar samples to facilitate the study of Hg biogeochemical processes at background sites with low anthropogenic influence. The setup utilizes the microwave-assisted digestion system for the digestion of a higher quantity of samples with the addition of a pre-digestion step. Results from analyzing foliar samples and CRMs showed that the proposed pre-concentration method is a robust and efficient way of preparing low Hg concentration samples for Hg isotopic analysis. Results from experiments with NIST SRM 3133, ^197^Hg radiotracer, and analysis on MC-ICP-MS validated the efficiency of pre-concentration by showing low Hg fractionation. In addition to its applicability for samples with biological origin having Hg low concentrations, this pre-concentration method can also be suitable for a variety of liquid samples with complex matrices as we observed an insignificant effect of the sample matrix on pre-concentration efficiency. Since the use of a microwave-assisted digestion system is a common practice for sample preparation, the optimized method proposed in this work would enable Hg pre-concentration for Hg isotope analysis in samples of different origins especially those having low Hg concentration.

### Supplementary information

Below is the link to the electronic supplementary material.

Supplementary file1 (DOCX 27 KB)
