# Supplementary material for: Optimization of a pre-concentration method for the analysis of mercury isotopes in low-concentration foliar samples
Source: Anal Bioanal Chem. 2024 Jan 9;416(5):1239–48. doi: 10.1007/s00216-023-05116-5 (PMC10850019; doi:10.1007/s00216-023-05116-5)
Supplement: Supplementary file 1 — Supplementary file1 (DOCX 27 KB) [file 216_2023_5116_MOESM1_ESM.docx]

# Appendix

Table S 1. Hg isotopic ratios measured in NIST SRM 1575a, NIST SRM 1547, NIST 3133 and NIST 8610. Values outside the parentheses correspond to the mean values whereas those inside the parentheses represent uncertainty expressed at k = 2.

| **Sample Name** | **Hg (ng/g)** | **δ^198^Hg** | **δ^199^Hg** | **δ^201^Hg** | **δ^202^Hg** | **δ^204^Hg** | **∆^199^Hg** | **∆^200^Hg** | **∆^201^Hg** | **∆^204^Hg** |
| --- | --- | --- | --- | --- | --- | --- | --- | --- | --- | --- |
| **NIST 8610** |  | -0.13 (0.02) | -0.25(0.08) | -0.43(0.12) | -0.52(0.15) | -0.77(0.17) | 0.00(0.02) | 0.01(0.02) | -0.04(0.03) | 0.01(0.09) |
| **NIST 3133** |  | -0.01(0.05) | 0.02(0.09) | 0.02(0.13) | 0.03(0.15) | 0.08(0.23) | -0.02(0.06) | 0.00(0.03) | 0.00(0.06) | 0.04(0.11) |
| **NIST SRM 1575a** | 39.9 | -0.59(0.12) | -0.58(0.15) | -1.22(0.21) | -1.16(0.20) | -1.73(0.34) | -0.32(0.08) | 0.02(0.04) | -0.35(0.07) | -0.01(0.07) |
| **NIST SRM 1547** | 31.7 | -0.57(0.01) | -0.85(0.02) | -1.47(0.00) | -1.72(0.02) | -2.58(0.17) | -0.13(0.01) | 0.01(0.01) | -0.17(0.01) | -0.01(0.14) |

Table S2. ^197^Hg radiotracer experiment recoveries for test with pure reagents.

| Exp | Recovery % | Leftover % | Residuals | Mass balance |
| --- | --- | --- | --- | --- |
| 1 | 99.7 | 1.2 | -0.3 | 100.9 |
| 2 | 99.4 | 0.7 | -0.6 | 100.1 |
| 3 | 96.8 | 0.6 | -3.2 | 97.4 |
| 4 | 98.7 | 0.7 | -1.3 | 99.4 |
| 5 | 98.3 | 0.4 | -1.7 | 98.8 |
| 6 | 96.8 | 0.7 | -3.2 | 97.4 |
| 7 | 98.2 | 0.3 | -1.8 | 98.5 |
| 8 | 98.3 | 0.6 | -1.7 | 98.9 |
| 9 | 102.4 | 1.2 | 2.4 | 103.6 |
| 10 | 97.7 | 0.2 | -2.3 | 97.9 |
| 11 | 100.9 | 0.4 | 0.9 | 101.2 |
| 12 | 98.3 | 0.3 | -1.7 | 98.6 |
| 13 | 100 | 0.4 | 0 | 100.4 |
| 14 | 98.8 | 1.1 | -1.2 | 99.9 |
| 15 | 101.6 | 0.4 | 1.6 | 102 |
| 16 | 96.1 | 0.8 | -3.9 | 96.9 |

Table S3. ^197^Hg radiotracer experiment recoveries for test with the addition of sample matrix.

| Exp. | Recovery % | Leftover % | Residuals | Mass balance |
| --- | --- | --- | --- | --- |
| 1 | 100.3 | 0.7 | 0.3 | 101.1 |
| 2 | 98.5 | 3.1 | -1.5 | 101.6 |
| 3 | 102.5 | 0.7 | 2.5 | 103.2 |
| 4 | 105.5 | 0.7 | 5.5 | 106.3 |
| 5 | 94.6 | 1.2 | -5.4 | 95.8 |
| 6 | 95.5 | 6.4 | -4.5 | 102 |
| 7 | 101.8 | 0.03 | 1.8 | 101.8 |
| 8 | 101.6 | 0.98 | 1.6 | 102.6 |
| 9 | 104 | 0.6 | 4 | 104.7 |
| 10 | 100.6 | 2.4 | 0.6 | 103 |
| 11 | 101.7 | 0.7 | 1.7 | 102.4 |
| 12 | 99.3 | 2.8 | -0.7 | 102.1 |
| 13 | 99.9 | 0.6 | -0.1 | 100.5 |
| 14 | 99.8 | 1.9 | -0.2 | 101.7 |
| 15 | 96.1 | 1.9 | -3.9 | 98 |

Table S4. NIST 3133 pre-concentration recoveries with pure reagents.

| Experiment No. | Hg added (ng) | Hg Captured (ng) | Estimated losses (ng) | Recovery (%) |
| --- | --- | --- | --- | --- |
| Test 1 | 16.27 | 16.20 | 0.07 | 99.55 |
| Test 2 | 16.27 | 15.29 | 0.98 | 93.96 |
| Test 3 | 16.27 | 15.51 | 0.76 | 95.34 |
| Test 4 | 16.27 | 15.17 | 1.10 | 93.22 |
| Test 5 | 16.27 | 15.14 | 1.13 | 93.06 |
| Test 6 | 16.27 | 14.75 | 1.52 | 90.68 |
| Test 7 | 16.27 | 15.49 | 0.78 | 95.21 |
| Test 8 | 16.27 | 15.41 | 0.85 | 94.75 |
| Test 9 | 16.27 | 16.07 | 0.20 | 98.76 |
| Test 10 | 16.27 | 15.81 | 0.46 | 97.19 |
| Test 11 | 32.54 | 30.63 | 1.90 | 94.15 |
| Test 12 | 32.54 | 30.98 | 1.56 | 95.21 |
| Test 13 | 32.54 | 29.69 | 2.85 | 91.26 |
